# Enlargement of the Uterus Cured by Iodine

**Published:** 1829-01-01

**Authors:** 


### XXVI.

### CASE OF INDURATED ENLARGEMENT OF THE UTERUS SUCCESSFULLY TREATED BY IODINE. By R. ASHBURNER THETFORD, M. D. &c.

[Dublin Transactions, Vol. V.]

Dr. Thetford was requested to visit a female on the 25th April, 1827, who was represented as being dangerously ill. She complained of great debility, and stated that, with the exception of a fever, she never had any illness. She was constipated in her bowels, and required immense doses of Epsom salts, (℥iv.) to open them. She had had no evacuation for *seventeen days*, though large doses of medicine had been taken. Vomiting—dysuria.

"She complained of headach, slight intolerance of light, and total want of sleep. Eyes dull, but not suffused; tongue coated with a yellowish white fur, and moist; inappetence; collapse of countenance; pulse 108, and very feeble; abdomen enlarged, and tender on pressure, particularly in the epigastric and hypogastric regions, in the latter of which pain was also felt. There was not much emaciation. She was in her fortieth year, and had borne one child when she was about sixteen years old. Additional symptoms indicated mechanical obstruction, and directed my attention to the vagina; but pain prevented further examination.

By the use of enemata the bowels began to act about two o'clock on the morning of the 27th, and a great quantity of very small scybala, with much offensive matter, came away, each evacuation causing considerable pain. The discharge from the bowels was preceded by convulsions, and frequent attacks of syncope. Oily aperients were afterwards taken daily, which produced several offensive and unnatural stools, attended with much pain, but general relief; and nightly anodynes were given to procure sleep. Under this treatment her general appearance and strength improved; but the tenderness and pain in the hypogastric region continued, also the pain in passing urine, which still came away in small quantities at a time. The pubic bones were very sensitive to the touch, and a tumour was felt above the symphysis.

"After some hesitation she now acknowledged that there was a hard tumour in the vagina, easily felt by herself, and very painful to the touch, which had gradually increased in size, without any discharge from it; and that menstruation had ceased for some months.

"I prescribed various sedatives to assuage pain and procure sleep, and directed the aperients to be continued until the fæcal discharges had become natural.

"This last object being obtained, I commenced an accurate local examination on the 9th of May. The attempt to introduce a finger into the vagina was met immediately within the labia by a projecting substance, which was easily ascertained to be the os uteri, somewhat enlarged, and firmer than natural; beyond, and connected with which, a large tumour of osseous hardness opposed resistance. I succeeded in introducing a portion of the index finger between it and the cavity of the sacrum, but laterally and anteriorly, any introduction between it and the other bones of the pelvis was impracticable. There could not exist a doubt of the tumour being the uterus of great size, and in a state of induration. I ordered alterative doses of the corrosive muriate of mercury, with pills of conium and hyosciamus, and enjoined a strict attention to the state of the bowels."

The above course was persevered in for 36 days; and, although the health was amended, the size and hardness of the uterus remained undiminished. On the 14th of June, Dr. Thetford prescribed seven drops of the tincture of iodine to be taken three times a day in a glass of water, which dose was augmented gradually to ten drops, every other medicine being discontinued, except castor oil occasionally.

"The result was speedily favourable; her spirits revived; appetite greatly improved; intestinal evacuations in general sufficient without any aperient; urine passed in greater quantity at a time, and with diminished pain; ability to take increased exercise; rapidly progressive absorption of the diseased substance of the uterus; periodical return of the catamenia.

"On the 2d of August she was perfectly restored to health, and continues so.

"It may be proper to state, that this individual was a patient of St. Thomas's Parish Dispensary, and unable to aid the effects of medicine by requisite diet."

If the above case was one in which the uterus was really of bony hardness, it affords a remarkable example of the power of iodine over the absorbent system. We believe, indeed, that no other medicine is possessed of equal power. The following is the form which we have found most effectual as an external application.

℞. Unguent. hydrargyri, ℥ss.

Axungiæ ............ ℥iss.

Hydriodatis potassæ, ℈ij.

Liq. potassæ......... gt. vj.

Misce fiat unguentum.

One drachm of this ointment rubbed daily on indolent glands and tumours will disperse them, if any application can do so.

---

